# Lipid and nutritional profiles of Caribbean patients with chronic kidney disease

**DOI:** 10.4314/ahs.v23i3.75

**Published:** 2023-09

**Authors:** Saleh Idris

**Affiliations:** Department of Chemical Pathology, Faculty of Clinical Sciences, Bayero University, Kano

**Keywords:** Cardiovascular diseases, chronic kidney disease, dyslipidemia, estimated glomerular filtration rate, kidney, malnutrition, parameter, risk factor

## Abstract

**Aim:**

Chronic kidney disease (CKD) is marked by the deterioration of kidney function and derangement in lipid metabolism. Thus, we aim at evaluating the lipid and nutritional parameters of Caribbean patients with CKD.

**Methods:**

The study recruited 88 CKD patients and 105 apparently healthy subjects. Blood glucose, urea, creatinine, albumin, uric acid, total cholesterol, triglycerides, HDL-cholesterol, VLDL, and LDL were measured in duplicate on the Vitros 4600 Multi-Channel Chemistry Auto-Analyzer (Johnson & Johnson Ortho-Clinical Diagnostics Inc., Rochester NY, USA) in our laboratory. The Statistical Package for the Social Sciences (SPSS, version 20) was used for statistical analysis.

**Results:**

Mean levels of diastolic blood pressure (p < 0.05), age, and systolic blood pressure between the patients and the healthy controls (p < 0.001) were different. In addition, mean levels of BUN (p < 0.05), serum creatinine, and uric acid were higher and eGFR lower in the patients compared with the healthy controls (p < 0.001). The mean levels of albumin, glucose, triglycerides, HDL-cholesterol, and VLDL (p < 0.001) also differed between patients and healthy controls. Negative correlation between eGFR and triglycerides and a positive correlation between eGFR and total cholesterol, HDL-c and LDL were observed. The prevalence of hypoalbuminemia, hypercholesterolemia and underweight were 27.27%, 57.95% and 4.55% respectively in patients, compared with 10.48%, 44.74% and 2.86% respectively in healthy controls.

**Conclusion:**

Dyslipidemia is common in CKD patients and is therefore, imperative that, routine lipid profile analysis be detailed in order to check any trend towards the development of CVD.

## Introduction

Chronic kidney disease (CKD) is a serious and common disease that results in multiple complications including premature mortality and end-stage kidney disease (ESKD) 1-3 with an estimated 1 in 7 to 10 adults worldwide having CKD. Only approximately 10% of these patients may likely survive to ESKD and only half of these survivors may likely have access to dialysis or kidney transplantation because of lack of access or due to high cost. 3 In addition, the prevalence of CKD has been noted to have increased between 1990 - 2016 by 90%, and related deaths mainly due to cardiovascular diseases and infections, nearly doubled in the United States and globally. 4-6 This increase in the prevalence of CKD could be closely related to the increase of at-risk populations with diabetes, hypertension, and pre-diabetes. Presently, the leading cause of CKD and global health emergency is diabetes with about 425 million individuals affected worldwide in 2017 and a projected 629 million individuals likely to be affected by 2045. 7-9

Hypertension is the second leading cause of CKD and affects nearly one-third of US adults and 1.13 billion people globally in 2015. 10-11 It has been estimated that, the global prevalence of CKD stands at 9.1% while the age-standardized global prevalence was reported to be higher in women and girls (9.5%) than in men and boys (7.3%). 12 However, about one third of all cases of CKD in the world were in China (132.3 million) and India (115.1 million), with 10 countries having >10 million cases and 79 countries having >1 million cases. In terms of mortality, CKD resulted in the death of 1.2 million people in 2017 and was reported to be the 12th leading cause of death worldwide. 12 Moreover, 7.6% of all cardiovascular diseases (CVD) deaths (1.4 million) could be attributed to impaired kidney function. In addition, there are still differences in CKD burden between countries and these differences are particularly apparent especially when comparing age-standardized CKD DALYs, which were apparently higher in Oceania, sub-Saharan Africa and Latin America. 12

The first manifestation of CKD is recognized by the presence of structural and /or functional damage to the kidney leading to a fall in the glomerular filtration rate (GFR) to <60 mLs/min for three months or more, regardless of the underlying cause. 13 Patho-physiologically, CKD is complex but mostly could be related to chronic derangement in metabolism resulting in disorders such as bone mineral diseases, increased cardiovascular disease (CVD) risk, glucose intolerance, acidosis, hyperuricemia and electrolyte abnormalities. 14

Risk factors associated with the development of the CKD include age, obesity, smoking, diabetes mellitus (DM) hypertension, and dyslipidemia. 15 Patients with CKD are at an increased risk for cardiovascular disease (CVD) and have a higher prevalence of hyperlipidemia than the general population. 16 Indeed, dyslipidemia, hypoalbuminemia and impaired glucose tolerance are features that fully described the adverse metabolic phenomena in CKD that leads to kidney damage and its progression. 17

Wanner et al., have suggested that hyperlipidemia could cause renal injury and contribute to the progression of renal disease. 18 Generally, the prevalence of hyperlipidemia increases as renal function declines, with the degree of hypertriglyceridemia and elevation of LDL cholesterol being proportional to the severity of renal impairment. 19 This is because mesangial cells express receptors for LDL and oxidized LDL, which upon activation induce me-sangial cell proliferation. In addition, macrophages infiltration may release cytokines which causes damage to the endothelial cells, mesangial cells and podocytes leading to progressive renal damage. 20 Studies have also shown that, lipid abnormalities are associated with a reduction in kidney function in the general population. 18

Moreover, a strong relationship between malnutrition, inflammation and atherosclerosis has been reported in CKD and this is sometimes referred to as “malnutrition-inflammation complex”. In this light, it has been suggested that, the rapid atherosclerosis occurring in advanced CKD might be mediated by synergism of different mechanisms including inflammation, malnutrition, oxidative stress and genetic components. 21-22 This complex involving inflammation and hypoalbuminemia are considered as a part of emerging (non-traditional) cardiovascular risk factors in CKD that contributes to cardiovascular disease, mortality and rapid progression to end stage kidney disease (ESKD). 23-24

The most typical and detrimental of the numerous complications of CKD however, is the progressive loss of body protein mass and energy reserves sometimes called protein-energy wasting (PEW). 25 This condition is more common in patients with advanced stages of CKD and may affect 18%-75% of patients with ESKD. (25-27) The condition could be caused by inadequate nutritional intake leading to malnutrition and could also results from an increased catabolism that is due to chronic low-grade inflammation leading to wasting. 25, 28 Thus we aim at evaluating the lipid and nutritional profiles of Caribbean patients with chronic kidney disease, a region that is presently witnessing rapid explosion in the population of people with CKD.

## Materials and methods

### Patient's recruitment

Data were retrieved and analysed from a study conducted between January and December 2016 which recruited 88 (55 males and 34 females) CKD patients of mean age 60.33 ± 1.35 and 105 (41 males and 64 females) apparently healthy controls of mean age 42.50 ± 1.34. All CKD patients were subjects attending the Nephrology Clinic of the Eric Williams Medical Science Complex (EWM-SC), Mount Hope, Trinidad and Tobago. The apparently healthy subjects were recruited from pools of civil servants and students' residents in St Augustine, Trinidad. An informed voluntary consent to participate in the study was obtained from all the subjects after the nature and benefits of participating in the study was explained to each.

### Eligibility criteria

All the patients recruited for the study have already been diagnosed with chronic kidney failure as established by an estimated glomerular filtration rate (GFR) of < 90 mLs/min/1.73m2 (calculated using the CKD-EPI equation). 29 Subjects excluded from the study are those on dialysis and those with acute kidney failure.

### Study protocol

Subjects were instructed to observed an overnight fast lasting between 10 – 12 hours after which five (5) millimetres of blood was collected from each. The blood was then transferred into a lavender top and a red top bottle. Plasma and sera were respectively separated and stored frozen at -80^o^C until laboratory analysis. Standard methods were used for the collection of anthropometric indices which included weight (measured in kilogram with clinic measuring scale), height (measured in meters with clinic measuring ruler). Other information such as age, gender, education, occupation, smoking history, alcohol consumption, and medical history including medications were also obtained. The blood pressure was measured for each subject after resting for about ten minutes using the dominant arm while the subject was in a sitting position. The study protocol was reviewed by the Ethics Committees of the University of the West Indies, St Augustine and North Central Regional Health Authority, Trinidad and approval was granted by these Ethical Committees.

### Laboratory analysis

All parameters including blood glucose, urea, creati-nine, albumin, uric acid, total cholesterol, triglycerides, HDL-cholesterol, and VLDL were all measured in duplicate on the Vitros 4600 Multi-Channel Chemistry Auto-Analyzer (Johnson & Johnson Ortho-Clinical Diagnostics Inc., Rochester NY, USA).

### Statistics and calculations

The data collected were analysed using the Statistical Package for the Social Sciences version 20 (SPSS Inc., 233 South Wacker Drive, Chicago, USA). Differences in categorical variables between groups were determined using the student t-tests while Chi-squared test was used for continuous variables. Differences in quantitative variables for the different patient groups were determined with ANOVA (with Bonferroni Post-Hoc analysis as appropriate) and p < 0.05 was considered statistically significant.

### Definitions

**Albumin:** Albumin level < 35g/L in this study is considered as low (hypoalbuminemia). 30

**Body Mass Index (BMI):** This was based on WHO categorization WHO criteria. 31

**Table:** 

BMI (kg/m^2^)	Nutritional Status
Below 18.5	Underweight
18.5 – 24.9	Normal weight
25.0 – 29.9	Pre-obesity
> 30.0 – 34.9	Obesity

### Risk factor definition

The European guidelines on cardiovascular diseases prevention in clinical practice 32 was use to established the optimum lipid levels in this study. High total cholesterol: > 4.5 mmol/L; high triglycerides: > 1.7 mmol/L and low HDL-C: < 1.0 mmol/L for males and < 1.2 mmol/L for females. 33

### CKD-EPI Equation

e-GFR = 141 x min (S_cr_/κ, 1) ^ά^ x max (S_cr_/κ, 1) - 1.209 x 0.993^Age^ x 1.018 [^if female^] x 1.159 [^if black^]

Where: S_cr_ is serum creatinine in mg/dl, ϰ is 0.7 for females and 0.9 for males, ἀ is -0.329 for females and -0.411 for males, min indicates the minimum of S_cr_/ϰ or 1, and max indicates the maximum of S_cr_/ϰ or 1 29.

### Chronic kidney disease categorization

The categorization was based on the Kidney Disease Outcome Quality Initiative (KDOQI) and Kidney Disease Improving Global Outcomes (KDIGO) recommendation. 34

**Table:** 

Stage		GFR	Description
1.		> 90 mL./min/1.73m^2^	normal kidney function but urine findings or structural abnormalities or genetic trait-point to kidney disease.
2.		60 – 89 mL./min/1.73m^2^	mildly reduced kidney function, and other findings (as for stage 1) point to kidney disease.
3A.		45 – 59 mL./min/1.73m^2^	moderately reduced kidney function.
3B.		30 – 44 mL./min/1.73m^2^	moderately reduced kidney function.
4.		15 – 29 mL./min/1.73m^2^	severely reduced kidney-function.
5.		< 15 mL./min/1.73m^2^	very severe, or end-stage kidney failure.

## Result

The results are expressed as mean (± SD) as appropriate. A total of 88 patients were recruited for the study. Table 1 shows there were differences in the mean levels of diastolic blood pressure (p < 0.05), age, systolic blood pressure and waist circumference between the patients and the healthy controls (p < 0.001). Compared to the controls, the mean levels of BUN (p < 0.05), serum creatinine, and uric acid (p < 0.001) were higher in the patients compared with the healthy controls, while eGFR was lower in patients compared with the healthy controls (p < 0.001; table 1).

**Table 1:** Age and Clinical Parameters of the CKD Patients and Healthy Controls

Parameter, Mean ± SD	CKD Patients (n = 88)	Controls (n = 105)
Age (yrs.)	60.33 ± 1.35	42.50 ± 1.34**
Height (m)	1.67 ± 0.01	1.66 ± 0.01
Weight (kg)	76.43 ± 2.05	75.11 ± 1.64
BMI (kg/m^2^)	27.37 ± 0.64	27.11 ± 0 .55
SBP (mmHg)	142.30 ± 2.61	122.45 ± 1.51**
DBP (mmHg)	81.82 ± 1.34	78.32 ± 1.11*
BUN (mmol/L)	13.89 ± 3.52	4.01 ± 0.14*
Creatinine (µmol/L)	223.55 ± 23.19	68.11 ± 1.73**
Uric Acid (µmol/L)	392.70 ± 14.70	289.12 ± 7.96**
eGFR (mLs/min/1.73m^2^)	48.44 ± 3.30	111.32 ± 1.99**

t-test

* p < 0.05

** p < 0.001

**Figure 1:**
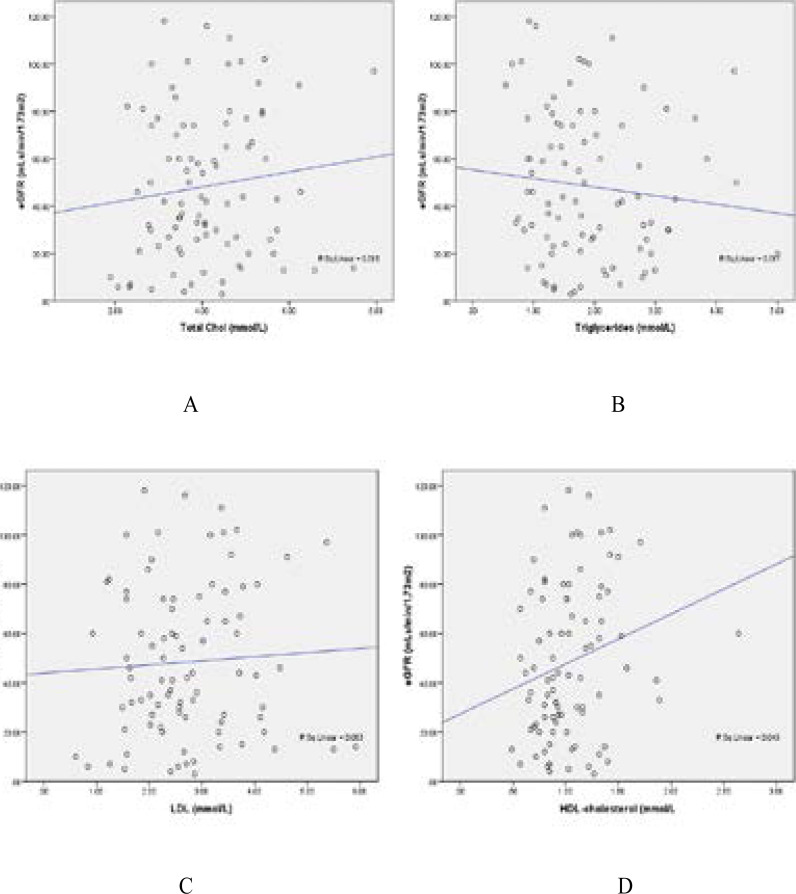
Correlation studies (r) between eGFR and some lipid parameters

Table 2 shows that, there are also differences in the mean levels of albumin, glucose, triglycerides, HDL-Cholesterol, and VLDL (p < 0.001) between the patients and healthy controls. Table 3 is the comparison of clinical parameters based on CKD staging while table 4 is the comparison of albumin, glucose and lipid parameters based on CKD staging. Figure 1 is the correlation studies between eGFR and some lipid parameters. Negative correlation was observed between eGFR and triglycerides while positive correlation was seen between eGFR and total cholesterol, HDL-c and LDL. Table 5 shows the prevalence of some nutritional parameters. While the prevalence of hypoalbuminemia, hypocholesterolemia and underweight were 27.27%, 57.95% and 4.55% among the patients respectively, the corresponding prevalence in healthy controls was lower and was 10.48%, 44.74 and 2.86% respectively.

**Table 2:** Albumin, Glucose and Lipid Profiles of the Patients and Healthy Controls

Parameter, Mean ± SD	CKD Patients	Controls
	(n = 88)	(n = 105)
Albumin (g/L)	36.97 ± 0.47	38.93 ± 0.36**
Glucose (mmol/L)	6.61 ± 0.31	4.31 ± 0.07**
T. Chol (mmol/L)	4.11 ± 0.13	4.16 ± 0.08
Triglycerides (mmol/L)	1.89 ± 0.10	1.24 ± 0.84**
HDL-C (mmol/L)	1.04 ± 0.04	1.21 ± 0.03**
VLDL (mmol/L)	0.38 ± 0.02	0.25 ± 0.02**
LDL (mmol/L)	2.71 ± 0.11	2.69 ± 0.08

t-test

* p < 0.05

** p < 0.001

**Table 3:** Comparison of Clinical Parameters of the Patients Based on CKD Stages

Parameter, Stage 5 Mean ± SD	CKD Stage 1	CKD Stage 2	CKD Stage 3	CKD Stage 4	CKD
Age (yrs.)	53.17 ± 1.28	59.10 ± 14.72*	63.59 ± 8.79*	63.08 ± 14.96*	60.87 ±
13.11*					
BMI (kg/m^2^)	27.59 ± 7.82	25.01 ± 5.80	27.60 ± 5.83	30.02 ± 5.69	26.11 ± 6.62
SBP (mmHg)	138.67 ± 1.66	137.67 ± 20.11	146.93 ± 24.11*	150.23 ± 36.61*	137.00 ± 22.04
DBP (mmHg)	81.92 ± 1.40	80.90 ± 8.95	83.62 ± 13.21	82.69 ± 14.16	78.75 ± 13.67*
BUN (mmol/L)	11.21 ± 2.16	6.62 ± 1.95*	10.15 ± 2.86	35.44 ± 82.45**	14.04 ± 5.48*
Scr (µmol/L)	83.19 ± 5.62	94.11 ± 14.64*	149.33 ± 30.18*	255.96 ± 54.70**	618.86 ±
258.29**					
UA (µmol/L)	283.38 ± 1.29	380.38 ± 11.39*	444.63 ± 86.60**	443.41 ± 17.70	356.69 ±
68.18*					
eGFR 10	101.58 ± 9.20	72.30 ± 8.25*	41.64 ± 9.28*	23.00 ± 3.79**	2.73 ± 1.56**
(mLs/min/1.73m^2^)					

t-test

* p < 0.05

** p < 0.001

**Table 4:** Comparison of Albumin, Glucose and Lipid Profiles of the Patients Based on CKD Stages

Parameter, Mean ± SD	CKD Stage 1	CKD Stage 2	CKD Stage 3	CKD Stage 4	CKD Stage 5
Albumin (g/L)	38.42 ± 3.80	37.75 ± 4.22	37.18 ± 4.19	36.23 ± 3.65	35.07 ± 5.87
Glucose (mmol/L)	6.15 ± 2.79	6.21 ± 2.54	6.73 ± 2.79	7.47 ± 4.07*	6.56 ± 2.93
T. Chol (mmol/L)	4.67 ± 1.45	4.03 ± 1.01	3.96 ± 0.89*	4.17 ± 0.99	4.00 ± 1.67
TG (mmol/L)	1.70 ± 1.07	1.83 ± 0.86	1.90 ± 0.95*	2.13 ± 1.06**	1.91 ± 0.67*
HDL-C (mmol/L)	1.20 ± 0.29	1.10 ± 0.43	1.04 ± 0.35*	0.86 ± 0.14*	0.99 ± 0.29*
VLDL (mmol/L)	0.34 ± 0.21	0.37 ± 0.17	0.38 ± 0.19	0.42 ± 0.21*	0.38 ± 0.13
LDL (mmol/L)	3.13 ± 1.13	2.56 ± 0.97*	2.54 ± 0.78*	2.89 ± 0.86*	2.73 ± 1.56*

t-test

* p < 0.05

** p < 0.001

**Table 5:** Prevalence of Nutritional Parameters among the Subjects Studied

Parameter, Mean ± SD	Categories of Subjects	
	CKD (n = 88)	Controls (n = 105)
Hypoalbuminemia (%)	27.27	10.48
Hypocholesterolemia (%)	57.95	44.74
Underweight (%)	4.55	2.86

## Discussion

Chronic kidney disease is marked by the deterioration of kidney function leading to the derangement of lipid parameters as the degree of kidney impairment progresses. The kidney thus become unable to regulate and excretes waste products of metabolism leading to their accumulation. Presently there is an increase in the global prevalence of CKD which could be attributed to the increase in the at-risk populations with diabetes, hypertension, and pre-diabetes 35-37. Reports have shown that, with every increase in the deterioration of kidney function, there is a corresponding rise in the accumulation of creatinine and other biochemical parameters 38. Thus, the data in table 3 of the present study indeed supports this assertion with the level of creatinine increasing while that of eGFR declining gradually with every increase in CKD staging. The implication of these is the retention of toxic waste products in the body of the CKD patients which may likely leads to toxicity and death. On the hand, the association between CKD and abnormalities in lipid metabolism and dyslipidemia has for long been established 39 and it was noted that, these lipid abnormalities do contribute significantly to the worsening renal functional decline 40.

Moreover, other risk factors for the progressive decline in renal function apparently interact with the unfavourable lipoprotein profiles as soon as renal function starts to decline 41. The commonest observed dyslipidemic pattern in the present study (Table 2) is that involving triglycerides, HDL-cholesterol (HDL-c) and very low-density lipoprotein (VLDL) and indeed, this pattern occurs gradually as the disease condition progresses (Table 4). This is the first documented report of dyslipidemia among CKD patients in Trinidad and Tobago. Similar observations were reported by Sabeela et al, 2014 42 in subjects from Karachi, Pakistan although their data showed derangement in triglycerides, HDL-cholesterol and LDL. It should nevertheless be noted that, dyslipidemia has previously been well recognized as an independent risk factor for cardiovascular disease (CVD) in non-renal subjects 43 and in particular, the direct atherogenic potential of the triglyceride-rich lipoproteins (TRLs), VLDLs and intermediate density lipoproteins (IDLs) 44.

In this light, studies have shown that VLDL and IDL accumulate in dialysis patients during kinetic turnover studies 45-47 and in obese chronic kidney disease (CKD) patients not yet on dialysis 48. In addition, HDL cholesterol dysfunction and LDL receptor-related protein (LRP) deficiency have also been reported and these may likely contribute to the increases in the chylomicron remnant and IDL cholesterol levels thereby providing another potential mechanism underlying the formation of small dense LDL (sdLDL) in CKD patients 49.

However, it should be noted that, despite the fact that, serum LDL cholesterol levels may remain within the normal range, the small dense low-density lipoprotein (sdLDL) (the most highly atherogenic subtype of LDL), could easily be oxidized thus leading to an increase in serum level as kidney function worsens 50. Therefore, both the IDL and sdLDL cholesterol can trigger the formation of atherosclerotic plaques even when the level of LDL cholesterol remained within the normal range 50-51. Thus, it is imperative that, routine detailed lipid profile analysis be perform in CKD patients as a way of checking the risk of development of CVD from dyslipidemia in these patients. Correlation studies between eGFR and some lipid parameters in the present study further add to the already established relationship between these parameters (figure 1). Indeed, previous studies have shown that, hyperlipidemia could cause renal injury and contribute to the progression of renal disease. 18

On the other hand, malnutrition a common finding among CKD patients is mostly diagnosed by serum albumin which is considered widely as a marker for nutritional status in CKD patients 52-53. Hence, serum albumin has been reported to be associated with poor outcome among CKD patients 54. In this study, we report that the prevalence of malnutrition of 27% observed in our patients was higher than the rate of 14% observed in the apparently healthy subjects (Table 5). However, this is the first documented report on the prevalence of malnutrition among CKD patients in Trinidad and Tobago. In addition, the prevalence of malnutrition reported in the present study is lower compared to the prevalence reported by previous studies 55-59. Several reasons could account for this observed difference.

First, renal replacement therapy (RRT) is free and widely available in Trinidad and Tobago. Patients therefore, utilized this modality frequently and have less frequency of dialysis skipping. This have helped tremendously in the reduction of factors which are the known major causes of malnutrition in CKD patients such as reduced food intake due to effect of uremia, reduced absorption of nutrients from edematous gut, metabolic acidosis, increased protein loss during dialysis especially peritoneal dialysis, inflammation, oxidative stress, carbonyl stress and hormonal disorders. 60-63

Secondly, previous studies might have used different criteria in establishing nutritional status in their patients including the use of tools such as BMI, subjective global assessment (SGA), or skin triceps thickness compared to serum albumin used in the present study. Moreover, we also report that, hypercholesterolemia is very common among the CKD patients in this study. Compared with a prevalence of 44% seen in healthy controls, CKD patients have a prevalence of 57%. Although some previous studies have reported similar findings, the finding is a source of concern especially bearing the fact hypercholesterolemia is well established risk factor for CVD among CKD patients and warrants the need for routine check of lipid profile among CKD patients in lifestyles clinics in Trinidad. This may help in the reduction in the risk of the development of CVD in CKD patients in the country.

## Conclusion

Dyslipidemia is common in CKD patients and is therefore, imperative that, routine lipid profile analysis be detailed in order to check trend in the development of CVD.
